# Supplementary material for: Emergence of Ceftazidime- and Avibactam-Resistant Klebsiella pneumoniae Carbapenemase-Producing Pseudomonas aeruginosa in China
Source: mSystems. 2021 Nov 2;6(6):e00787-21. doi: 10.1128/mSystems.00787-21 (PMC8562488; doi:10.1128/mSystems.00787-21)
Supplement: TABLE S1 [file msystems.00787-21-st001.docx]

Table S1 KPC-encoded *P. aeruginosa* replicons in NCBI

| Name | GenBank accession | Nation | Year | Host ST | Replicon |  |
| --- | --- | --- | --- | --- | --- | --- |
| pBH6 | CM003767 | Brazil | 2016 | 244 | plasmid |  |
| plasmid unnamed1 | CP027168 | USA | 2018 | NA | plasmid |  |
| plasmid unnamed2 | CP029092 | USA | 2018 | NA | plasmid |  |
| 24Pae112 | CP029605 | Colombia | 2019 | 235 | chromosome | |
| pBH9 | CP029714 | Brazil | 2018 | 381 | prophage | |
| plasmid p1 | CP040685 | China | 2019 | NA | plasmid | |
| pR31-KPC | CP061851 | China | 2020 | NA | plasmid | |
| pPA-2 | KC609322 | Colombia | 2013 | 1006 | plasmid | |
| pCOL-1 | KC609323 | Colombia | 2013 | 308 | plasmid | |
| p10265-KPC | KU578314 | China | 2016 | NA | plasmid | |
| pD5170990 | KX169264 | Brazil | 2016 | NA | plasmid | |
| p14057-KPC | KY296095 | China | 2018 | NA | plasmid | |
| p1011-KPC2 | MH734334 | China | 2019 | 463 | plasmid | |
| YLH6_p3 | MK882885 | China | 2019 | 463 | plasmid | |
| pPA2047 | MN082782 | Argentina | 2020 | NA | plasmid | |
| p4130-KPC | MN336501 | France | 2020 | NA | plasmid | |
| pNK546a | MN433457 | China | 2019 | 664 | plasmid | |
