# Supplementary material for: Emergence of Ceftazidime- and Avibactam-Resistant Klebsiella pneumoniae Carbapenemase-Producing Pseudomonas aeruginosa in China
Source: mSystems. 2021 Nov 2;6(6):e00787-21. doi: 10.1128/mSystems.00787-21 (PMC8562488; doi:10.1128/mSystems.00787-21)
Supplement: TABLE S3 [file msystems.00787-21-st003.docx]

**Table S3-1 Sample type comparison between KPC-PA and non-KPC-PA**

| **sample type** | **KPC-PA**  **(n=151)** | **non-KPC-PA**  **(n=223)** | **OR** | **95% CI** | **adjusted P value** |
| --- | --- | --- | --- | --- | --- |
| abdominal drainage | 25 | 11 | 3.81* | (1.74-8.89) | 0.09 |
| abscess | 8 | 13 | 0.90 | (0.31-2.42) | 1 |
| ascites | 4 | 4 | 1.50 | (0.27-8.12) | 1 |
| bile | 2 | 17 | 0.16 | (0.02-0.70) | 1 |
| blood | 20 | 44 | 0.62 | (0.33-1.14) | 1 |
| catheter | 4 | 2 | 3.00 | (0.42-33.54) | 1 |
| cerebrospinal fluid | 0 | 1 | 0 | (0-57.55) | 1 |
| lower respiratory tract | 7 | 29 | 0.33 | (0.12-0.79) | 1 |
| other drainage | 1 | 8 | 0.18 | (0.00-1.36) | 1 |
| pleural effusion | 5 | 1 | 7.56 | (0.83-360.50) | 1 |
| secretion | 1 | 6 | 0.24 | (0.00-2.02) | 1 |
| skin | 6 | 2 | 4.55 | (0.80-46.74) | 1 |
| sputum | 24 | 65 | 0.46 | (0.26-0.79) | 0.98 |
| upper respiratory tract | 4 | 5 | 1.19 | (0.23-5.61) | 1 |
| urine | 33 | 8 | 7.48* | (3.25-19.35) | <0.0001 |
| mediastinal drainage | 1 | 0 | Inf | (0.04-Inf) | 1 |
| stool | 5 | 0 | Inf | (1.37-Inf) | 1 |
| subdermal drainage | 1 | 0 | Inf | (0.04-Inf) | 1 |
| NA | 0 | 7 | 0 | (0-1.01) | 1 |

skin includes skin ulcer, abdominal incision, abdominal incision, burns, wound, cervical incision, and surgical incision; catheter includes hemodialysis catheter, central venous catheter, PICC, internal jugular vein catheter; Inf: infinite

**Table S3-2 Department comparison between KPC-PA and non-KPC-PA**

| **department** | **KPC-PA (n=151)** | **non-KPC-PA (n=223)** | **OR** | **95% CI** | **adjusted P value** | |
| --- | --- | --- | --- | --- | --- | --- |
| cardiology | 0 | 1 | 0 | (0-57.55) | 1 | |
| cardiothoracic surgery | 1 | 2 | 0.74 | (0.01-14.28) | 1 |  |
| emergency | 2 | 11 | 0.26 | (0.03-1.21) | 1 |  |
| emergency surgery | 0 | 1 | 0 | (0-57.55) | 1 |  |
| endocrinology | 0 | 1 | 0 | (0-57.55) | 1 |  |

| ENT | 1 | 2 | 0.74 | (0.01-14.28) | 1 |
| --- | --- | --- | --- | --- | --- |
| gastroenterology | 2 | 4 | 0.74 | (0.07-5.20) | 1 |
| general internal medicine | 0 | 1 | 0 | (0-57.55) | 1 |
| general surgery | 41 | 44 | 1.52 | (0.90-2.54) | 1 |
| geriatrics | 1 | 13 | 0.11 | (0.00-0.73) | 0.35 |
| hematology | 2 | 9 | 0.32 | (0.03-1.58) | 1 |
| hematology and oncology | 0 | 1 | 0 | (0-57.55) | 1 |
| ICU | 54 | 61 | 1.48 | (0.92-2.36) | 1 |
| infectious diseases | 1 | 5 | 0.29 | (0.01-2.64) | 1 |
| international department | 0 | 1 | 0 | (0-57.55) | 1 |
| nephrology | 0 | 1 | 0 | (0-57.55) | 1 |
| neurology | 2 | 11 | 0.26 | (0.03-1.21) | 1 |
| neurosurgery | 12 | 9 | 2.05 | (0.77-5.66) | 1 |
| oncology | 2 | 1 | 2.97 | (0.15-176.4) | 1 |
| oncosurgery | 0 | 1 | 0 | (0-57.55) | 1 |
| orthopedics | 2 | 6 | 0.49 | (0.05-2.77) | 1 |
| radiology | 0 | 1 | 0 | (0-57.55) | 1 |
| rehabilitation medicine | 8 | 2 | 6.15 | (1.20-60.33) | 0.59 |

| respiratory medicine | 7 | 16 | 0.63 | (0.21-1.67) | 1 |
| --- | --- | --- | --- | --- | --- |
| thoracic surgery | 1 | 1 | 1.48 | (0.02-116.6) | 1 |
| traditional Chinese medicine | 1 | 1 | 1.48 | (0.02-116.6) | 1 |
| trauma | 1 | 1 | 1.48 | (0.02-116.6) | 1 |
| urology clinic | 0 | 1 | 0 | (0-57.55) | 1 |
| vascular surgery | 1 | 6 | 0.24 | (0.01-2.02) | 1 |
| VIP | 0 | 1 | 0 | (0-57.55) | 1 |
| bone marrow transplantation | 1 | 0 | Inf | (0.04-Inf) | 1 |
| cardiosurgery | 1 | 0 | Inf | (0.04Inf) | 1 |
| urology | 5 | 0 | Inf | (1.37-Inf) | 0.35 |
| NA | 2 | 7 | 0.42 | (0.04-2.22) | 1 |

Inf: infinite
