# Supplementary material for: Emergence of Ceftazidime- and Avibactam-Resistant Klebsiella pneumoniae Carbapenemase-Producing Pseudomonas aeruginosa in China
Source: mSystems. 2021 Nov 2;6(6):e00787-21. doi: 10.1128/mSystems.00787-21 (PMC8562488; doi:10.1128/mSystems.00787-21)
Supplement: TABLE S4 [file msystems.00787-21-st004.docx]

Table S4. Primers used in this study

| Primers | sequences (5'-3') | Efficiency | R^2^ |
| --- | --- | --- | --- |

| *bla*_KPC_ screening |  |  |  |  |
| --- | --- | --- | --- | --- |
| KPC-2_FW | TTCTGCTGTCTTGTCTCTC |  |  | |
| KPC-2_RV | AGGTTCCGGTTTTGTCTC |  |  |  |
| qPCR |  |  |  |  |
| rpoD-qF | CTTACGCGGAGGTCAACGAC | 104.69% | 0.9945 |  |
| rpoD-qR | TCCGGGGCTGTCTCGAATAC |  |  |  |
| ampC-qF | CATCGGCCTGTTCGGCTATC | 103.51% | 0.9885 |  |
| ampC-qR | TCGAGGTGGGTCTGTTCGAG |  |  |  |
| mexB-qF | CGGCAGCATGACCAAGGAAG | 108.11% | 0.9982 |  |
| mexB-qR | ACACCTGGAAGTCACCGACG |  |  |  |
| KPC-qF | CGCCGTCTAGTTCTGCTGTC | 109.18% | 0.9964 |  |
| KPC-qR | CCGCCAAAGTCCTGTTCGAG |  |  |  |
| Plasmid curing check |  |  |  |  |
| trbM_FW | GTTGAGCGTCTGCAAGTCAG |  |  |  |
| trbM_RV | GAACCACACGAACCAGGCT |  |  |  |
