# Supplementary material for: Emergence of Ceftazidime- and Avibactam-Resistant Klebsiella pneumoniae Carbapenemase-Producing Pseudomonas aeruginosa in China
Source: mSystems. 2021 Nov 2;6(6):e00787-21. doi: 10.1128/mSystems.00787-21 (PMC8562488; doi:10.1128/mSystems.00787-21)
Supplement: TABLE S5 [file msystems.00787-21-st005.docx]

**GenBank accession numbers of 233 chromosomes and 97 plasmids to construct a customized Kraken2 library**

| Chromosome | Plasmid |
| --- | --- |
| NC_002516.2 | NZ_CP015000.1 |
| NC_008463.1 | NZ_CP016215.1 |
| NC_009656.1 | NZ_CP018048.1 |
| NC_011770 | NZ_CP017294.1 |
| NC_017548.1 | NZ_CP020561.2 |
| NC_017549.1 | NZ_CP020561.1 |
| NC_018080.1 | NZ_CP020602.1 |
| NC_020912.1 | NZ_CP024631.1 |
| NC_021577.1 | NZ_CP025052.1 |
| NC_022806.1 | NZ_CP025054.1 |
| NC_022808.1 | NZ_CP027170.1 |
| NC_022808.2 | NZ_CP027167.1 |
| NC_023019.1 | NZ_CP027168.1 |
| NC_023066.1 | NZ_CP027173.1 |
| NC_023149.1 | NZ_CP027175.1 |
| NZ_AAQW01000001.1 | NZ_CP027176.1 |
| NZ_AFXJ01000001.1 | NZ_CP029094.1 |
| NZ_AFXK01000001.1 | NZ_CP029091.1 |
| NZ_AP014622.1 | NZ_CP029092.1 |
| NZ_AP014646.1 | NZ_CP029096.1 |
| NZ_AP014651.1 | NZ_CP029095.1 |
| NZ_AP014839.1 | NZ_CP029708.1 |
| NZ_AP017302.1 | NZ_CP030914.1 |
| NZ_CP007147.1 | NZ_CP032256.1 |
| NZ_CP007399.1 | NZ_CP033772.1 |
| NZ_CP008739.1 | NZ_CP033773.1 |
| NZ_CP008856.1 | NZ_CP033834.1 |
| NZ_CP008856.2 | NZ_CP034355.1 |
| NZ_CP008857.1 | NZ_CP039294.1 |
| NZ_CP008858.1 | NZ_CP039989.1 |
| NZ_CP008858.2 | NZ_CP039991.1 |
| NZ_CP008859.1 | NZ_CP040126.1 |
| NZ_CP008859.2 | NZ_CP040685.1 |
| NZ_CP008860.1 | NZ_CP041355.1 |
| NZ_CP008860.2 | NZ_CP042268.1 |
| NZ_CP008861.1 | NZ_LS998784.1 |
| NZ_CP008862.1 | NZ_LT969519.1 |
| NZ_CP008862.2 | NZ_LT969521.1 |
| NZ_CP008863.1 | CP029714 |
| NZ_CP008864.1 | CP045003 |
| NZ_CP008864.2 | CP049162 |
| NZ_CP008865.1 | KP873171 |
| NZ_CP008865.2 | KP873172 |
| NZ_CP008866.1 | KP975076 |
| NZ_CP008866.2 | KR106190 |
| NZ_CP008867.1 | KR106191 |
| NZ_CP008868.1 | KU254577 |
| NZ_CP008869.1 | KU578314 |
| NZ_CP008869.2 | KX169264 |
| NZ_CP008870.1 | KX709966 |
| NZ_CP008870.2 | KX711879 |
| NZ_CP008871.1 | KX889311 |
| NZ_CP008871.2 | KY296095 |
| NZ_CP008872.1 | KY296096 |
| NZ_CP008872.2 | KY494864 |
| NZ_CP008873.1 | KY630469 |
| NZ_CP010555.1 | LGVH01000782 |
| NZ_CP011317.1 | LN809998 |
| NZ_CP011857.1 | MF141039 |
| NZ_CP012001.1 | MF144194 |
| NZ_CP012066.1 | MF168945 |
| NZ_CP012578.1 | MF344568 |
| NZ_CP012579.1 | MF344569 |
| NZ_CP012580.1 | MF344570 |
| NZ_CP012581.1 | MF344571 |
| NZ_CP012582.1 | MF344578 |
| NZ_CP012583.1 | MF344579 |
| NZ_CP012584.1 | MH053445 |
| NZ_CP012585.1 | MH061383 |
| NZ_CP012679.1 | MH463250 |
| NZ_CP012901.1 | MH547560 |
| NZ_CP013113.1 | MH547561 |
| NZ_CP013245.1 | MH594579 |
| NZ_CP013477.1 | MH734334 |
| NZ_CP013478.1 | MK047609 |
| NZ_CP013479.1 | MK047610 |
| NZ_CP013696.1 | MK882885 |
| NZ_CP013989.1 | MN082782 |
| NZ_CP013993.1 | MN386974 |
| NZ_CP014866.1 | MN433456 |
| NZ_CP014948.1 | MN433457 |
| NZ_CP014999.1 | MN583270 |
| NZ_CP015001.1 | NC_007100 |
| NZ_CP015002.1 | NC_008357 |
| NZ_CP015003.1 | NC_009739 |
| NZ_CP015117.1 | NC_010722 |
| NZ_CP015377.1 | NC_016138 |
| NZ_CP016214.1 | NC_020452 |
| NZ_CP016955.1 | NC_022344 |
| NZ_CP017099.1 | NC_022345 |
| NZ_CP017099.1 | NC_022346 |
| NZ_CP017149.1 | NZ_CM007350 |
| NZ_CP017293.1 | NZ_CM017760 |
| NZ_CP017306.1 | NZ_CM019124 |
| NZ_CP017353.1 | NZ_CP011370 |
| NZ_CP017969.1 | NZ_CP043482 |
| NZ_CP019338.1 | NZ_CP043548 |
| NZ_CP020560.1 |  |
| NZ_CP020560.1 |  |
| NZ_CP020603.1 |  |
| NZ_CP020659.1 |  |
| NZ_CP020703.1 |  |
| NZ_CP020704.1 |  |
| NZ_CP021774.1 |  |
| NZ_CP021775.1 |  |
| NZ_CP021999.1 |  |
| NZ_CP022000.1 |  |
| NZ_CP022001.1 |  |
| NZ_CP022002.1 |  |
| NZ_CP022478.1 |  |
| NZ_CP022525.1 |  |
| NZ_CP022526.1 |  |
| NZ_CP023255.1 |  |
| NZ_CP023316.1 |  |
| NZ_CP024477.1 |  |
| NZ_CP024630.1 |  |
| NZ_CP025049.1 |  |
| NZ_CP025050.1 |  |
| NZ_CP025051.1 |  |
| NZ_CP025053.1 |  |
| NZ_CP025055.1 |  |
| NZ_CP025056.1 |  |
| NZ_CP026680.1 |  |
| NZ_CP027165.1 |  |
| NZ_CP027166.1 |  |
| NZ_CP027169.1 |  |
| NZ_CP027171.1 |  |
| NZ_CP027172.1 |  |
| NZ_CP027174.1 |  |
| NZ_CP027538.1 |  |
| NZ_CP028132.1 |  |
| NZ_CP028162.1 |  |
| NZ_CP028584.1 |  |
| NZ_CP028584.2 |  |
| NZ_CP028848.1 |  |
| NZ_CP028849.1 |  |
| NZ_CP028917.1 |  |
| NZ_CP028959.1 |  |
| NZ_CP029088.1 |  |
| NZ_CP029089.1 |  |
| NZ_CP029090.1 |  |
| NZ_CP029093.1 |  |
| NZ_CP029097.1 |  |
| NZ_CP029605.1 |  |
| NZ_CP029660.1 |  |
| NZ_CP029707.1 |  |
| NZ_CP029745.1 |  |
| NZ_CP030327.1 |  |
| NZ_CP030328.1 |  |
| NZ_CP030351.1 |  |
| NZ_CP030861.1 |  |
| NZ_CP030910.1 |  |
| NZ_CP030911.1 |  |
| NZ_CP030912.1 |  |
| NZ_CP030913.1 |  |
| NZ_CP031449.1 |  |
| NZ_CP031449.2 |  |
| NZ_CP031659.1 |  |
| NZ_CP031660.1 |  |
| NZ_CP031677.1 |  |
| NZ_CP032126.1 |  |
| NZ_CP032257.1 |  |
| NZ_CP032552.1 |  |
| NZ_CP032569.1 |  |
| NZ_CP032761.1 |  |
| NZ_CP033432.1 |  |
| NZ_CP033439.1 |  |
| NZ_CP033684.1 |  |
| NZ_CP033685.1 |  |
| NZ_CP033686.1 |  |
| NZ_CP033771.1 |  |
| NZ_CP033832.1 |  |
| NZ_CP033833.1 |  |
| NZ_CP033835.1 |  |
| NZ_CP033843.1 |  |
| NZ_CP034354.1 |  |
| NZ_CP034368.1 |  |
| NZ_CP034369.1 |  |
| NZ_CP034409.1 |  |
| NZ_CP034429.1 |  |
| NZ_CP034434.1 |  |
| NZ_CP034435.1 |  |
| NZ_CP034436.1 |  |
| NZ_CP035739.1 |  |
| NZ_CP037925.1 |  |
| NZ_CP037926.1 |  |
| NZ_CP039293.1 |  |
| NZ_CP039988.1 |  |
| NZ_CP039990.1 |  |
| NZ_CP040127.1 |  |
| NZ_CP040684.1 |  |
| NZ_CP041008.1 |  |
| NZ_CP041013.1 |  |
| NZ_CP041354.1 |  |
| NZ_CP041771.1 |  |
| NZ_CP041772.1 |  |
| NZ_CP041773.1 |  |
| NZ_CP041774.1 |  |
| NZ_CP041945.1 |  |
| NZ_CP042269.1 |  |
| NZ_CP043328.1 |  |
| NZ_CP044006.1 |  |
| NZ_CP045739.1 |  |
| NZ_CP045768.1 |  |
| NZ_CP046060.1 |  |
| NZ_CP046061.1 |  |
| NZ_CP046069.1 |  |
| NZ_CP047592.1 |  |
| NZ_CP047697.1 |  |
| NZ_LN870292.1 |  |
| NZ_LN871187.1 |  |
| NZ_LR130527.1 |  |
| NZ_LR130528.1 |  |
| NZ_LR130530.1 |  |
| NZ_LR130531.1 |  |
| NZ_LR130533.1 |  |
| NZ_LR130534.1 |  |
| NZ_LR130535.1 |  |
| NZ_LR130536.1 |  |
| NZ_LR130537.1 |  |
| NZ_LR134308.1 |  |
| NZ_LR134309.1 |  |
| NZ_LR134330.1 |  |
| NZ_LR134342.1 |  |
| NZ_LR590472.1 |  |
| NZ_LR590473.1 |  |
| NZ_LR590474.1 |  |
| NZ_LR657304.1 |  |
| NZ_LS998783.1 |  |
| NZ_LT608330.1 |  |
| NZ_LT673656.1 |  |
| NZ_LT883143.1 |  |
| NZ_LT969520.1 |  |
